# Identifying effect modifiers of CAR-T cell therapeutic efficacy: a systematic review and individual patient data meta-analysis protocol

**DOI:** 10.1186/s13643-022-02158-1

**Published:** 2023-01-19

**Authors:** Manoj M. Lalu, Natasha Kekre, Joshua Montroy, Maryam Ghiasi, Kevin Hay, Scott McComb, Risini Weeratna, Harold Atkins, Brian Hutton, Ayel Yahya, Ashish Masurekar, Mohamad Sobh, Dean A. Fergusson

**Affiliations:** 1 Centre for Practice-Changing Research, Office L1298a, Clinical Epidemiology Program, Ottawa Hospital Research Institute, 501 Smyth Road, Box 201B, Ottawa, Ontario K1H 8L6 Canada; 2 Department of Anesthesiology and Pain Medicine, The Ottawa Hospital, Ottawa, Canada; 3 Blood and Marrow Transplant Program, Ottawa Hospital Research Institute, Ottawa, Canada; 4 Department of Medicine, University of British Columbia, Vancouver, Canada; 5 National Research Council of Canada, Ottawa, Canada; 6 Division of Medicine, Department of Hematology, University of Ottawa, Ottawa, Canada; 7 School of Epidemiology and Public Health, University of Ottawa, Ottawa, Canada

**Keywords:** Systematic review, Individual patient data meta-analysis, CAR-T cell therapy, Oncology, Protocol

## Abstract

**Background:**

Chimeric antigen receptor T cell therapy (CAR-T) represents a promising and exciting new therapy for hematologic malignancies, where prognosis for relapsed/refractory patients remains poor. Encouraging results from clinical trials have often been tempered by heterogeneity in response to treatment among patients, as well as safety concerns including cytokine release syndrome. The identification of specific patient or treatment-specific factors underlying this heterogeneity may provide the key to the long-term sustainability of this complex and expensive therapy. An individual patient data meta-analysis (IPMDA) may provide potential explanations for the high degree of heterogeneity. Therefore, our objective is to perform a systematic review and IPDMA of CAR-T cell therapy in patients with hematologic malignancies to explore potential effect modifiers of CAR-T cell therapy.

**Methods and analysis:**

We will search MEDLINE, Embase, and the Cochrane Central Register of Controlled Clinical Trials. Studies will be screened in duplicate at the abstract level, then at the full-text level by two independent reviewers. We will include any prospective clinical trial of CAR-T cell therapy in patients with hematologic malignancies. Our primary outcome is complete response, while secondary outcomes of interest include overall response, progression-free survival, overall survival, and safety. IPD will be collected from each included trial and, in the case of missing data, corresponding authors/study sponsors will be contacted. Standard aggregate meta-analyses will be performed, followed by the IPD meta-analysis using a one-stage approach. A modified Institute of Health Economics tool will be used to evaluate the risk of bias of included studies.

**Ethics and dissemination:**

Identifying characteristics that may act as modifiers of CAR-T cell efficacy is of paramount importance and can help shape future clinical trials in the field. Results from this study will be submitted for publication in a peer-reviewed scientific journal, presented at relevant conferences and shared with relevant stakeholders.

**Supplementary Information:**

The online version contains supplementary material available at 10.1186/s13643-022-02158-1.

## Introduction

Patients with CD19+ hematologic malignancies have a very poor prognosis in the relapsed or refractory setting. For example, patients with relapsed diffuse large B cell lymphoma after autologous stem cell transplant have a median survival of 10 months [1], and patients with acute lymphoblastic leukemia that relapse after allogeneic stem cell transplant have a 1-year survival under 20% [2]. Chimeric antigen receptor (CAR) T cell therapy is now widely accepted as treatment for these patients. Molecularly modified T cells from either the donor or recipient can be genetically engineered to express a CAR (e.g., via retrovirus) that detects a specific antigen associated with tumors. Thus, the T cells are able to locate and kill cells expressing that antigen (e.g., CD19, CD30, etc.). Phase I and phase II clinical trials using CAR-T cells have been carried out for patients with relapsed or refractory hematologic malignancies [3–6]. These studies and others have reported continued remissions [7–9].

Although most clinical trials using CD19 CAR-T cells have generally been relatively small, evidence to date has demonstrated durable responses, suggesting that many patients who respond are cured of their underlying malignancy [7–9]. These encouraging results have been tempered by a lack of efficacy for some patients as well as safety considerations, such as cytokine release syndrome (CRS), neurotoxicity, and other less common adverse events such as clinical anaphylaxis and immune effector cell–associated neurotoxicity syndrome [ICANS] [10, 11]. Further refinement of CAR-T cell therapy will require identifying specific patient or treatment characteristics and their impact on treatment response and adverse events remain unknown such as sex, age, treatment dose, and co-stimulatory domain of the CAR.

Our previous systematic review explored the efficacy and safety of CAR-T cell therapy in patients with any form of malignancy [12]. Sixty studies were included in the review (42 studies of patients with hematologic malignancies). Despite 54% complete response rates in patients with CD19+ hematologic malignancies, the variability in efficacy across trials was considerable. While a priori defined subgroups analyses demonstrated higher levels of complete response in acute lymphoblastic leukemia patients, as well as CAR-T products constructed using a 4-1BB co-stimulatory domain, the presence of significant heterogeneity in effect sizes remained unexplained. In addition, there were a multitude of important factors which we could not explore due to limitations of the traditional, study-level data driven approach. For example, the age of patients included in individual trials ranged from early twenties to late seventies. Effects across age, however, could not be assessed with the aggregate, study-level data approach. Similarly, dosing regimens were heterogeneous not only between studies, but within studies themselves (e.g., inconsistent dosing among patients in the same study), and varied from 2 × 10^5^ to 1 × 10^10^ cells/kg. Using individual patient data (IPD) meta-analyses may present an opportunity to examine patient-level characteristics which may modify the effect of CAR-T cell therapy.

In the last two decades, the use of IPD has become increasingly popular as it provides many statistical and clinical (i.e., identifying important subgroups of interest) advantages over traditional, aggregate-level data meta-analyses [13]. IPD meta-analyses involve the analysis of individual-level data rather than the aggregate-level data used in traditional meta-analyses. There are several advantages to this approach [14, 15], with the ability to identify effects within important subgroups (i.e., clinical or intervention characteristics) being one of the significant advantages [16]. Using an IPD approach, it becomes possible to model individual-level interactions directly within studies. This affords greater power and reduces ecological bias when compared to subgroup analyses or meta-regression that incorporates aggregate data from different studies [17]. In the past, an IPD approach has helped refine future trial eligibility criteria, based on results of important subgroup analyses [18, 19]. In fact, a recent IPD meta-analysis explored the relationship between severe CRS and CAR-T dose and demonstrated that severe CRS was positively associated with increasing infusion doses. However, this review did not explore parameters of CAR-T efficacy and collected limited baseline patient variables [20].

Therefore, we believe an IPD approach has the potential to identify important subgroups of patients that may be more responsive to the therapeutic efficacy of CAR-T cell therapy. Our objective is to conduct a systematic review and individual patient data meta-analysis of CAR-T cell therapy in patients with hematologic malignancies to explore potential effect modifiers of CAR-T cell therapy.

## Protocol

This systematic review protocol follows the Preferred Reporting Items for Systematic reviews and Meta-Analysis Protocol (PRIMSA-P) guideline (see Additional file 1) [21]. In addition, the protocol has been registered on the International Prospective Registry of Systematic Reviews PROSPERO (Registration number CRD42021205552).

### Eligibility criteria

#### Population

We will include studies in patients with hematologic malignancies. Studies in patients with solid tumor malignancies will not be included. In the previous systematic review of CAR-T cell therapy by members of our group, there was no signal for efficacy in studies of solid tumor malignancies (complete response of 4.1 %, 95% CI, 1.6 to 10.6%) [12]; therefore, the decision was made to focus on studies of hematologic malignancy.

#### Intervention

Our intervention is chimeric antigen receptor T cell therapy. There will be no restrictions based on therapeutic dose, the number of therapeutic administrations, CAR-T target, CAR-T co-stimulatory domain, etc.

#### Comparator

Studies with or without comparators will be included.

#### Outcomes

The primary outcome of interest is complete response. Secondary outcomes include overall response, disease progression, relapse, overall survival, and adverse events (cytokine release syndrome, neurotoxicity, B cell aplasia, graft-versus-host disease). Tertiary outcomes of interest include patient experience, health-related quality of life, and health utility measures.

#### Study design

We will include all interventional studies. Observational studies (i.e., retrospective cohort studies, case report, case series, etc.) will be excluded.

### Information sources

Searches will be conducted in the MEDLINE (OVID interface, including In-Process and Epub Ahead of Print), Embase (OVID interface), and the Cochrane Central Register of Controlled Trials (Wiley interface) databases from October 2017 to present. This represents the date at which the search from our previous review was last run [12]. We will update the search before publishing the full systematic review.

### Search strategy

The literature search used in our previous review will be used as a guide [12]. The field of CAR-T cell therapy is rapidly evolving, and therefore new search terms will need to be developed and added to the search. This new search will be developed in conjunction with an information specialist and will be specific to hematologic malignancy patients. Prior to the execution of the full search strategy, a Peer Review of Electronic Search Strategies (PRESS) will be performed [22]. This represents an additional layer of methodological rigor, helping to ensure the accuracy of our search strategy. We will also search reference lists of included studies and relevant reviews identified in our search. The systematic review team will then be provided with a bibliography of included articles for feedback. A sample search strategy can be found in the online supplement (Additional file 2).

### Study selection process

Results of the literature search will be uploaded to Distiller Systematic Review Software (DistillerSR®, Evidence Partners, Ottawa). The titles and abstracts will be independently reviewed by two reviewers using the predefined inclusion criteria as described above. To refine the screening question and to establish a high level of inter-rater correlation (kappa > 0.8), a calibration test involving ten studies will be performed before screening begins formally. The full text will be accessed for all titles that meet the inclusion criteria or in case of uncertainty. Two review authors will assess the full reports for eligibility. A third-party member will be consulted in order to resolve disagreements. The reasons for excluding studies will be recorded. For non-English full text articles, we will contract Ottawa Hospital faculty or employees with fluency in the language of the article to assist in determining article eligibility. The study selection process will be documented and reported using the Preferred Reporting Items for Systematic Reviews and Meta-analyses (PRISMA) flow diagram.

### Data extraction process

Data extraction forms will be developed in DistillerSR® by the study team in order to capture all relevant information from each included study. Two reviewers will extract all relevant data from the included studies using this extraction form. Five studies will be used for calibration, and the review team will also be consulted for guidance if the data extraction form needs to be revised. Any disagreements will be reconciled through consensus and, if necessary, a third party will make a final decision. Where uncertainty is identified, or data of interest is not reported, study authors will be contacted for more information.

### Data items and individual patient data extraction

We will collect basic study characteristics (i.e., first author, year of publication, country of trial, trial design, sample size), study-level patient data (sex, age, comorbidities, cancer type, previous lines of treatment, treatment dose, follow-up period, etc.), and intervention characteristics will be similar to our previous review [12] (manufacturing and cell product characteristics, including fresh or frozen, T cell origin, selection of T cell subsets, T cell expansion method including cell culture duration, CAR target antigen, CAR molecular structure [e.g., affinity domain, hinge domain, transmembrane domain, costimulatory domain(s), signaling domain], transfection/transduction method and the therapeutic regimen [CAR-T cell dose, frequency, duration, route of administration]). Outcome data and risk of bias information (both described below) will also be extracted. In addition, we will collect the same information listed above but at the individual patient level.

### Dealing with obtaining IPD when it is not reported

The process of obtaining not-reported IPD can be lengthy and complex [23, 24], and failure to include IPD within an IPDMA can compromise the review’s objective, decrease statistical power, and introduce potential biases [25, 26]. Therefore, significant effort will be made to obtain not-reported IPD. Our review team will contact the corresponding author and/or study sponsor for each included clinical trial where IPD is not reported. We will follow recently published practical guidance on obtaining and managing individual patient data datasets [27]. In the case of non-response or refusal, data from these clinical trials will be included in the study-level analysis only. A complete list of all the data items listed above will be sought from each included clinical trial. Upon identification of not-reported IPD from each trial, it will be clearly outlined to corresponding authors what data we are asking for. If corresponding authors express interest in collaborating and providing data, they will be invited to read the protocol, and a virtual meeting will be scheduled to discuss details of sharing IPD. If IPD is provided, the corresponding author of the trial will be invited to be listed as a collaborator on the manuscript. Any data provided will be anonymized and stored on a secured server. Any remaining IPD that we fail to obtain will not be imputed and will be classified as missing. Also, if there is missing data for patients, we will classify them as missing and not impute for missing values.

### Outcomes

Our primary outcome is complete response. Complete response definitions will vary, given our broad range of malignancies being included in the review. Complete response definitions will be collected. We will report the best overall response if a complete response is not possible. Complete response data will not include studies involving patients who were in complete remission at the time they started CAR-T cell therapy. The complete response data from the latest timepoint will be used. For example, we anticipate the identification of multiple publications from singular trials (i.e., longer-term follow-up). In this case, complete response rates at the longest follow-up provided will be extracted and analyzed. The duration of the complete response will be collected. Secondary outcomes include overall response, progression-free survival, relapse, overall survival, and adverse events (cytokine release syndrome, neurotoxicity, B cell aplasia, graft-versus-host disease). Overall response will be defined as the sum of partial and complete responses. Tertiary outcomes of interest include patient experience, health-related quality of life, and health utility measures.

### Risk of bias assessment

We will use a modified version of the Institute of Health Economics tool to assess risk of bias [28, 29]. This modified tool was successfully implemented in previous reviews consisting of predominately single arm studies [12, 30]. As we have previously described, the tool evaluates the following elements: study objective, design, study population, intervention and cointerventions, outcome measures, statistical analysis, results, and conclusions, as well as conflicts of interest. The risk of bias for any controlled or comparative studies will be evaluated using the appropriate risk of bias assessment tool for randomized or non-randomized comparative trials recommended by Cochrane [31]. Risk of bias details will be extracted in duplicate by two independent reviewers, with any disagreements being resolved by a third party.

### Data analysis

#### Study-level analysis

Our data analysis will consist of two approaches. First, we will perform the traditional, aggregate data approach. Briefly, dichotomous data (i.e., complete response) will be analyzed and presented in a proportion (truncated between 0 and 1) with 95% confidence intervals. Using Comprehensive Meta-Analysis (version 3; Biostat Inc., USA), we will use a random-effects model (DerSimonian and Laird) to pool outcomes. The Cochrane *I*^2^ statistic will be used to assess statistical heterogeneity. Alternative funnel plots (specific to single-arm studies) will be used to assess publication bias [32]. The quality of evidence will be assessed by GRADE (Grading of Recommendations, Assessment, Development and Evaluations) [33].

#### Individual patient data meta-analysis

Second, we will perform an IPDMA, which will be used to explore the effect of patient-level characteristics on our identified outcome measures. For the IPDMA, a one-stage approach will be used, which synthesizes the IPD from all included studies in a single step, while simultaneously accounting for the clustering of patients within studies [13]. Specifically, we will use a generalized linear mixed model with reduced random intercept and random slope which assumes the random effects to be independent. The one-stage approach was chosen, as it allows for more sophisticated modeling to explore our effect moderators of interest as opposed to pooling trial level estimates. By using this method, the exact binomial distribution is modeled, correlations within the study are taken into account which allows for greater flexibility in the model specification, and continuity corrections are not required [34]. A sensitivity analysis will be conducted using the two-stage approach, to assess the robustness of our analysis. The investigation of patient-level factors which may be acting as effect modifiers are described below in the subgroup analysis section.

#### Subgroup analyses

Next, we will perform subgroup analyses at the patient-level, as subgroup exploration at the patient-level is the primary purpose of our study. We will explore the effects of patient-level covariates on the efficacy (complete response) and safety (adverse events) of CAR-T cell therapy. Covariates of interest include sex, age, cancer type, cancer stage, previous number of treatments, use of lymphodepletion, use of bridging therapy, follow-up period, duration of response, CAR-T dose, number of CAR-T cell infusions, time between leukapheresis and infusion, CAR-T source (allogeneic vs autologous), CAR-T formulation, and co-stimulatory domain used. These covariates of interest have been chosen based on findings from our previous systematic review of CAR-T cell therapy, as well as discussions with clinical experts on the review team [12, 35]. Cancer type and CAR-T formulation are considered core covariates and will be included in all modeling. For assessing interactions, we will undertake a one-stage meta-analysis with a generalized linear model to estimate treatment-covariate interaction with both random treatment and interaction effects. The assumptions of such a model are that the true effect of the treatment varies between studies, the true effect of the interaction varies between studies, and that the random effects for both treatment and interaction are uncorrelated. Study-level subgroup analyses will be performed in the event that a significant portion of IPD is unable to be obtained. Study-level subgroup analyses include type of malignancy, age group (pediatric or adult), T cell origin (autologous or allogeneic), and CAR-T construct (e.g., CD19 or CD20, etc.).

### Ethics and dissemination

Institutional ethics approval is not required for this study. Results from this review will be published in a peer-reviewed scientific journal and presented at appropriate scientific conferences. The final reporting of this manuscript will follow the guidance set forth by the Preferred Reporting Items for Systematic Reviews and Meta-Analyses IPD statement [36]. Finally, our diverse team of collaborators and knowledge users will aide with the dissemination of study findings. In order to maximize impact of our study results, we have assembled a nationally representative team of knowledge-users. The Leukemia & Lymphoma Society of Canada (LLSC) has partnered with our team on prior CAR-T related research projects [37]. This partnership proved fruitful and they have once again agreed to act as a knowledge user for this proposed project. Cell Therapy Transplant Canada (CCTC) is a national organization within Canada, representing physicians, allied healthcare providers, and researchers working in the fields of hematopoietic cell transplantation and cellular therapy. They also have a diverse network of patients and patient’s families and are expertly positioned to act as knowledge users for this proposed research. Prior to study start-up, we will have a teleconference between team members and knowledge users to summarize our protocol and make any final adjustments based on reviewer comments. We will also identify any further stakeholders that may be interested. At the commencement of each new step in the project (i.e., screening, data extraction, etc.), we will have teleconference meetings to track progress. To meet the needs of our knowledge users, feedback will be continually sought in order to ensure that stakeholders are contributing in a meaningful manner with respect to the design and execution of our two major objectives including whether they feel their input is being heard and integrated.

### Patient and public involvement

In developing this protocol, patients and members of the public were not involved. However, we have an ongoing relationship with two patient partners for CAR-T research, and we plan to involve our patient partners going forward in the project, specifically around the interpretation and disseminate of results. Our group has had a fruitful relationship with patient partners in the past for CAR-T-related research [37], involving them at many different stages of various projects.

## Discussion

The results from our project will have the potential to shape future treatment and research in the field of CAR-T cell therapy. The heterogeneity seen in efficacy both between and within clinical trials has been a detriment to the advancement of the field of CAR-T cell therapy as the variability in patient and therapeutic characteristics limited our ability to draw certain conclusions and to evaluate the impact of important clinical and intervention characteristics such as patient sex and age or dosing characteristics. By using individual patient data, as opposed to data aggregated at the study level, we are increasing our ability to identify important characteristics which may be modifying the efficacy of CAR-T cell therapy. The impacts of IPD meta-analyses on trial design can range from comparator selection, to population and outcome definition [18, 19], to the creation of stopping rules [38]. The results from our proposed project may help identify subgroups of patients who respond optimally to CAR-T cell therapy. Given the safety and cost concerns, it is important that the right group of patients be properly identified and treated safely.

## Supplementary Information

**Additional file 1.** PRISMA-P (Preferred Reporting Items for Systematic review and Meta-Analysis Protocols) 2015 checklist.

**Additional file 2.** Sample Search Strategy.

## Data Availability

Open Science Framework: Preferred Reporting Items for Systematic review and Meta-Analysis Protocols (PRISMA-P) checklist and search strategy for ‘Identifying Effect Modifiers of CAR-T Cell Therapeutic Efficacy: A Systematic Review and Individual Patient Data Meta-Analysis Protocol’. [DOI 10.17605/OSF.IO/XVRJ7] Data are available under the terms of the CC-By Attribution 4.0 International. No other data are associated with this article.
