# Supplementary material for: Identifying effect modifiers of CAR-T cell therapeutic efficacy: a systematic review and individual patient data meta-analysis protocol
Source: Syst Rev. 2023 Jan 19;12:9. doi: 10.1186/s13643-022-02158-1 (PMC9850506; doi:10.1186/s13643-022-02158-1)
Supplement: Supplementary file 2 — Additional file 2. Sample Search Strategy. [file 13643_2022_2158_MOESM2_ESM.pdf]

Additional File 2. Sample Search Strategy
Database: Ovid MEDLINE(R) ALL
Search Strategy:
----
1 ((chimeric antigen adj2 receptor*) and (therap* or treat* or immunity or immunotherap* or cell*)).tw,kw.
2 ((car adj3 t adj5 therap*) or (car adj3 t adj5 treat*)).tw,kw.
3 (car adj3 t adj3 immunotherap*).tw,kw.
4 Receptors, Antigen, T-Cell/tu
5 (car therap* or (car adj2 t adj2 cell*)).tw,kw.
6 ((modified or engineered) adj2 (t cell* or t lymphocyte*)).tw,kw.
7 Receptors, Antigen, T-Cell/ and (Adoptive Transfer/ or Immunotherapy, Adoptive/ or Immunotherapy/)
8 Receptors, Chimeric Antigen/ and Immunotherapy, Adoptive/
9 car t.tw,kw.
10 (axicabtagene ciloleucel or tisagenlecleucel).mp. or axi cel.tw,kw. or tisa cel.tw,kw.
11 or/1-10
12 exp Lymphoma/ or exp Leukemia/ or exp Hematologic Neoplasms/ or exp Multiple Myeloma/
13 (h?ematolog* cancer* or lymphoid malignanc* or b cell malignan* or h?ematolog* neoplasm* or h?ematolog* malignanc* or lymphoma* or leuk?emi* or myeloma* or nonhodgkin* or non hodgkin* or t cell malignan*).tw,kw.
14 12 or 13
15 11 and 14
16 (2017112* or 2017113* or 201712* or 2018* or 2019* or 2020*).dt.
17 15 and 16
18 (2021* or 20201*).dt.
19 17 not 18

Database: Embase Classic+Embase
Search Strategy:
----
1 ((chimeric antigen adj2 receptor*) and (therap* or treat* or immunity or immunotherap* or cell*)).tw.
2 ((car adj3 t adj5 therap*) or (car adj3 t adj5 treat*)).tw.
3 (car adj3 t adj3 immunotherap*).tw.
4 (car therap* or (car adj2 t adj2 cell*)).tw.
5 ((modified or engineered) adj2 (t cell* or t lymphocyte*)).tw.
6 chimeric antigen receptor/
7 tisagenlecleucel T/
8 axicabtagene ciloleucel/
9 (axicabtagene ciloleucel or tisagenlecleucel or axi cel or tisa cel).tw.
10 car t.tw.
11 chimeric antigen receptor t-cell immunotherapy/
12 or/1-11

13 (h?ematolog* cancer* or lymphoid malignanc* or b cell malignan* or h?ematolog* neoplasm* or h?ematolog* malignanc* or lymphoma* or leuk?emi* or myeloma* or nonhodgkin* or non hodgkin* or t cell malignan*).tw.
14 hematologic malignancy/ or exp lymphoma/ or exp leukemia/ or exp multiple myeloma/
15 13 or 14
16 12 and 15
17 conference abstract.pt.
18 conference.so.
19 17 or 18
20 16 not 19
21 (2017112* or 2017113* or 201712* or 2018* or 2019* or 2020*).dc.
22 20 and 21
23 20201*.dc.
24 22 not 23

Database: EBM Reviews - Cochrane Central Register of Controlled Trials
Search Strategy:
----
1 ((chimeric antigen adj2 receptor*) and (therap* or treat* or immunity or immunotherap* or cell*)).tw,kw.
2 ((car adj3 t adj5 therap*) or (car adj3 t adj5 treat*)).tw,kw.
3 (car adj3 t adj3 immunotherap*).tw,kw.
4 Receptors, Antigen, T-Cell/tu
5 (car therap* or (car adj2 t adj2 cell*)).tw,kw.
6 ((modified or engineered) adj2 (t cell* or t lymphocyte*)).tw,kw.
7 Receptors, Antigen, T-Cell/ and (Adoptive Transfer/ or Immunotherapy, Adoptive/ or Immunotherapy/)
8 car t.tw,kw.
9 (axicabtagene ciloleucel or tisagenlecleucel).mp. or axi cel.tw,kw. or tisa cel.tw,kw.
10 Receptors, Chimeric Antigen/ and Immunotherapy, Adoptive/
11 or/1-10
12 exp Lymphoma/ or exp Leukemia/ or exp Hematologic Neoplasms/ or exp Multiple Myeloma/
13 (h?ematolog* cancer* or lymphoid malignanc* or b cell malignan* or h?ematolog* neoplasm* or h?ematolog* malignanc* or lymphoma* or leuk?emi* or myeloma* or nonhodgkin* or non hodgkin* or t cell malignan*).tw,kw.
14 12 or 13
15 11 and 14
16 limit 15 to yr="2017 -Current"
